# Anlotinib inhibits growth of human esophageal cancer TE-1 cells by negative regulating PI3K/Akt signaling pathway

**DOI:** 10.1007/s12672-024-00995-1

**Published:** 2024-04-27

**Authors:** Yueli Liu, Fan Li, Qiongyu Wang, Yunfei Zhang, Shuhong Tian, Biao Li

**Affiliations:** 1 Department of Pharmacology, Hainan Medical University, Haikou, China; 2 Department of Thoracic Surgery, The Second Affiliated Hospital of Hainan Medical University, No. 368 Yehai Avenue, Haikou, China; 3 Department of Biostatistics, New York University, Jersey City, NJ USA; 4 Research Center for Drug Safety Evaluation of Hainan, Hainan Medical University, No. 3 Xueyuan Road, Haikou, China

**Keywords:** Anlotinib, Apoptosis, Cell Cycle, Esophageal cancer, PI3K/Akt signaling pathway

## Abstract

Anlotinib is effective in treatment of many kinds of malignant cancer, but its antineoplastic effects on esophageal cancer remains unclear. This study aims to investigate its impact on esophageal cancer and the underlying mechanisms. Anlotiniband 5-fluorouracil + cisplatin (5-FU + DDP) was administered separately to human esophageal cancer TE- 1 cells tumor xenograft mouse models every 3 days. Tumor size and body weight were measured before each treatment and at the end of the experiment. In vitro studies were conducted using TE- 1 cells to examine the effects of Anlotinib. Cell viability, migration, proliferation, apoptosis, cell cycle, their regulatory proteins and the transcriptomic changes were analyzed. Anlotinib reduced tumor size, tumor weight, and the ratio of tumor weight to body weight in vivo. It decreased the viability of TE- 1 cells, with a 50% growth-inhibitory concentration of 9.454 μM for 24 h, induced apoptosis, and arrested TE- 1 cell cycle in the S phase. It inhibited migration and proliferation while negatively regulating the PI3K/Akt signaling pathway. Enhanced expressions of P21, Bax, and lowered expressions of cyclin A1, cyclin B1, CDK1, PI3K, Akt, p-Akt, and Bcl-2 were observed after Anlotinib treatment. Anlotinib exhibits antineoplastic activity against human esophageal cancer TE- 1 cells by negatively regulating the PI3K/Akt signaling pathway, consequently altering the expressions of proteins related to proliferation, apoptosis, and the cell cycle.

## Introduction

Esophageal cancer (EC) is characterized by high morbidity and mortality [1, 2]. It consists of two major histologic subtypes: adenocarcinoma and squamous cell carcinoma(SCC), with SCC being the predominant subtype. EC exhibits significant regional variations globally. Certain regions in Asia, East Africa, and South America, such as China, Iran, Kenya, and Brazil, have higher incidence rates of esophageal cancer. This is associated with local factors such as diet, lifestyle, cooking methods, food choices, and environmental factors. Despite significant advancements in surgery, preoperative chemotherapy, and radiotherapy, the survival rate remains low, with a 5-year survival rate ranging from 15 to 20%, and a median survival time of 1.5 years [3]. This is despite the considerable progress in surgery, preoperative chemotherapy, and radiotherapy. The therapy regimen primarily depends on the physical condition and tumor stage, typically classified by the TMN stage of EC patients. Chemotherapy is widely applied to patients who are not suitable for surgery.

Anlotinib is a recently approved chemotherapeutic agent that has gained approval for the treatment of advanced non-small-cell lung cancer as a third-line therapy option in China [4, 5]. It functions as a multikinase inhibitor, targeting vascular endothelial growth factor receptors (EGFR) 1–3, fibroblast growth factor receptors 1–4, the platelet-derived growth factor receptor, and the stem cell factor receptor to inhibit neoangiogenesis and tumor progression [6–8]. Anlotinib also inhibits the activity of basic fibroblast growth factor receptor (bFGFR). bFGFR is another crucial receptor associated with tumor growth and angiogenesis. By affecting the bFGFR signaling pathway, Anlotinib can regulate cell growth, differentiation, and migration, thereby influencing the development of tumors. Anlotinib has been found to induce apoptosis in tumor cells, which refers to programmed cell death. This is an essential part of the normal cell lifecycle but is often disrupted in tumors. By prompting tumor cells to undergo programmed cell death, Anlotinib helps inhibit the growth of tumors.

Furthermore, studies have demonstrated the efficacy of anlotinib against intrahepatic cholangiocarcinoma [9], soft tissue sarcoma [10], thyroid cancer [11], and colorectal cancer [12]. It exhibited notable therapeutic effects against esophageal squamous cell carcinoma (ESCC) on patient-derived xenograft models when combined with chemoradiotherapy, while the exact mechanism remains unclear. Therefore, our study was designed to investigate the efficacy and underlying mechanism of anlotinibon ESCC using tumor xenograft animal models and TE- 1 cells.

## Methods

### Tumor xenograft mouse model

All animals’ procedures were approved by Institutional Animal Care and Use Committee of Research Center for Drug Safety Evaluation of Hainan province (China) and all animal experiments took place at Research Center for Drug Safety Evaluation of Hainan, Hainan Medical University. Male nude BALB/c mice were used to establish the TE- 1 xenograft model. TE- 1 cells were harvested and adjusted a cell density of 1 × 10^7^ per mL with PBS, and then 0.1 mL of cell suspension was injected subcutaneously in the right armpit area of each mouse. The diameters of length and width were measured to calculate tumor size. The formula: V = 0.5 × (length × width2) mm3 was used to calculate tumor volume (mm3). When the tumor volume grew to 100–300 mm3, atotal of 23 mice with weights in the range of 18–22 g were selected randomly and subdivided into control (n = 8), 5-fluorouracil (5-FU) + cisplatin (DDP) (n = 8) and anlotinib (n = 7) groups. Anlotinib was given intragastrically in a dose of 3 mg/kg; 5-FU and DDP were administered into the caudal vein in doses of 5 mg/kg and 20 mg/kg respectively on day 0. Sterile saline was used as the control. The drugs were administered every 3 days for 5 times. The body weight and tumor sizes were measured before each administration and in the end of the experiment. On day 15, all mice were sacrificed by inhalation of carbon dioxide. Tumor tissues were isolated and weighed, and then the ratio of tumor weight to body weight was calculated.

### Cell culture

The human-derived TE- 1 cell line and normal esophageal epithelial cell line (HET‑1A) were purchased from the Cell Bank of the Chinese Academy of Sciences (Shanghai, China). The TE- 1 cells were cultured in 1640 basal medium (Gibco, Waltham, MA, USA, Cat No: 8118131) supplemented with 10% fetal bovine serum (Gibco, Waltham, MA, USA, Cat No: 42F7180K); the HET‑1A cells were cultured in complete growth medium. The cells were cultured in a humidified atmosphere of 5% CO_2_ at 37 ℃. Cells in the logarithmic growth phase were utilized for study.

### MTT assay

TE-1 and HET‑1A cells were meticulously seeded into individual wells of a 96-well plate, with a standardized count of 8000 cells per well to ensure consistency across experiments. Anlotinib, obtained from Sigma-Aldrich (St. Louis, MO, USA), was initially dissolved in dimethyl sulfoxide (DMSO) and then further diluted in basal medium to achieve a range of concentrations spanning from 0.01 to 10 μM. Following the preparation of the drug solutions, the cells were subjected to treatment with each concentration of anlotinib for a predetermined duration of 24 h to allow for adequate drug exposure. Subsequently, 20 μL of MTT solution (5 mg/mL, purchased from aladdin, Cat No: G1724034) was carefully added to each well, followed by an additional incubation period of 4 h. After completion of the incubation, the supernatant containing excess MTT solution was meticulously aspirated, and 150 μL of DMSO was added to each well in a light-protected environment to dissolve the formazan crystals formed by viable cells. The plate was then placed on a plate agitator and shaken for 10 min to ensure thorough dissolution. Finally, the optical density of the resulting solution was measured at 550 nm using a microplate reader to quantify the cellular metabolic activity indicative of cell viability. Each experiment was rigorously replicated three times to ensure the reliability and reproducibility of the results. The percentage of cell viability was calculated by averaging the values obtained from the three independent replications, providing a robust assessment of the cellular response to anlotinib treatment.

### Flow cytometry

The TE-1 cell density was adjusted to 1 × 10^5^ per mL, and then samples were placed in a 6-well plate with 1 × 10^5^ cells per well. Cells were cultured for 48 h and treated with anlotinib in doses of 5 μM and 10 μM for another 24 h. Cells were digested with 0.25% trypsin without EDTA and then centrifuged (3000 rpm, 20 ℃, 3 min). The cells were washed 3 times for 2 min each time and suspended in 0.5 mL of PBS.

Cell apoptosis assay: A commercial annexin V-FITC/propidium iodide (PI) apoptosis detection kit (BD, Cat No: 556547) was used to analyze cell apoptosis. Cells were washed twice with cold PBS, and then were suspended and adjust to 1 × 10^6^ per mL with buffer. 100 μL cell suspension was added with 5 μL of annexin V-FITC and 5 μL of PI and then incubated for 15 min at 25 ℃ in darkness according to the instruction of manufacturer. Cell apotosis was analyzed by flow cytometry within 1 h.

Cell cycle assay: The cells were fixed with 75% alcohol at 4 ℃ for 12 hand then centrifuged (1000 rpm, 20 ℃, 3 min). After discarding supernatant, the cells were washed and then supplemented with PBS to adjust the cell density to 1 × 10^5^ per mL. The cells were incubated with 0.5 mL of PI (BD, Cat No: 550825) at 25 ℃ for 30 min in darkness. Cell cycle was analyzed by flow cytometry within 1h. All experiments were replicated three times.

### Wound closure assays

In our experiment, we followed rigorous steps for wound closure analysis. Firstly, we seeded 4 × 10^5^ cells in each well of a 6-well plate, ensuring they were in logarithmic growth phase with seeding efficiency ranging between 80 to 90%. During the critical growth phase of cells, wounds were precisely mechanically created using a 10 μL pipette tip, which is crucial for standardizing the wound creation process and ensuring consistency across all samples. Post-wound creation, cells were immediately treated with three different doses of Alectinib (2.5 μM, 5 μM, and 10 μM). Throughout the experiment timeline, specifically at 24 and 48 h post-treatment, meticulous documentation was maintained. High-resolution images of the wounds were captured at designated time points using appropriate imaging equipment. Subsequently, precise analysis was performed using Image J software, including accurate measurements of the wound area to determine the percentage of wound closure. This step was repeated thrice to ensure robustness and reliability of our results. Through repetition of experiments, our aim was to reduce experimental variability and enhance the statistical power of the results.

### Transwell assays

After TE-1 cells were treated with anlotinib for 48h, the cells were digested and resuspended in a concentration of 2.5 × 10^5^/mL with basal 1640 medium. 200 μL suspension was added to the upper chambers with 8.0 µm membrane pores and cells were allowed to migrate toward the bottom chambers, which contained 500 μL basal 1640 medium with 10% FBS for 24 h. Then the cells on the underside of the filter membrane were fixed in 4% paraformaldehyde for 20 min and stained with 0.1% crystal violet (Solarbio, Cat No: G1063) for 20 min. The migrated cells were photographed and counted under microscope, 5 views were chosen with 4 views from surrounding and 1 view from center of filter membrane and the migrated cell counts were used for analysis.

### Colony formation assays

After treated with anlotinib in doses of 2.5, 5, 10 μM for 48 h, TE- 1 cells were seeded with a concentration of 150 cells/well and cultured a humidified atmosphere of 5% CO_2_ at 37 ℃. Cells culture was terminated when the cell colony can be seen with unaided eyes. Subsequently, the cell colonies were fixed in 4% paraformaldehyde for 20 min and then stained with 0.1% crystal violet for 20 min at room temperature. The stained colonies were then imaged and counted for further analysis.

### Transcriptome assay

TE- 1 cells were treated with anlotinib (3, 10 μM) or 5-FU (3 μM) + DDP (3 μM) for 24 h. DMSO was used as a control. Total RNA was extracted by using a Trizol reagent kit (Invitrogen, Carlsbad, CA, USA, Cat No: G3013). The mRNA was enriched with oligo (dT) beads and then cleaved into short fragments, which were reverse transcribed into cDNA with random primers. Second-strand cDNA was synthesized with RNase H, DNA polymerase I, dNTP, and buffer. The cDNA fragments were then processed by purification with a QiaQuick PCR extraction kit (Qiagen, Venlo, The Netherlands, Cat No: G3322), end repair, poly (A) addition, and ligation to illumine sequencing adapters. The ligation products were selected according to their size by agarose gel electrophoresis, PCR amplified, and sequenced. Finally, the PCR products were subjected to an Illumina HiSeq2500 system from Gene Denovo Biotechnology Co.(Guangzhou, China).

### Western blot assays

After treated with anlotinib in a dose of 10 μM or 5-fluorouracil (5-FU, 3 μM) + cisplatin (DDP, 3 μM) for 12 h, the TE- 1 cell was lysed in RIPA lysis buffer (Servicebio, Cat No: G2002) to extract protein. Then the protein was quantified and measured by using a BCA kit (Servicebio, Cat No: G2026) and SDS-PAGE (Servicebio, Cat No: G2003). 10 μg proteins per well were loaded on to the gel for immunoblotting. Polyvinylidene difluoride membranes (Servicebio, Cat No: G6015-0.45) were blocked with 5% nofat milk in TBST buffer, incubated with primary antibodies (1:1000) targeting Bax (Servicebio, Cat No: GB11690), Bcl-2 (Servicebio, Cat No: GB113375), P21(Servicebio, Cat No: GB113721), cyclin-dependent protein kinase 1(CDK1, Servicebio, Cat No:GB11398), cyclin A1(Servicebio, Cat No: GB113964), cyclin B1(Servicebio, Cat No: GB112098), survivin (Servicebio, Cat No: GB11177), Akt(Servicebio, Cat No: GB111114), phosphorylated-Akt (p-Akt, Servicebio, Cat No: AF0908), and PI3K (Servicebio, Cat No: GB112375) at 4°Covernight, and then incubated HRP-conjugated secondary antibody (1:5000) for 30 min at 25 ℃. The signals were visualized by using an ECLkit (Servicebio, Cat No: G2014). Image Pro software was employed to calculate the intensity of the signals in each sample.

### Statistical Methods

All data are expressed as the mean ± the SD. SPSS 23.0 software was employed to perform the statistical analysis. Intergroup comparisons were studied by using attest or one-way ANOVA; P values less than 0.05 were considered statistically significant.

## Results

### Anlotinib exhibits anticancer activity in tumor xenograft mouse models

**Fig. 1:**
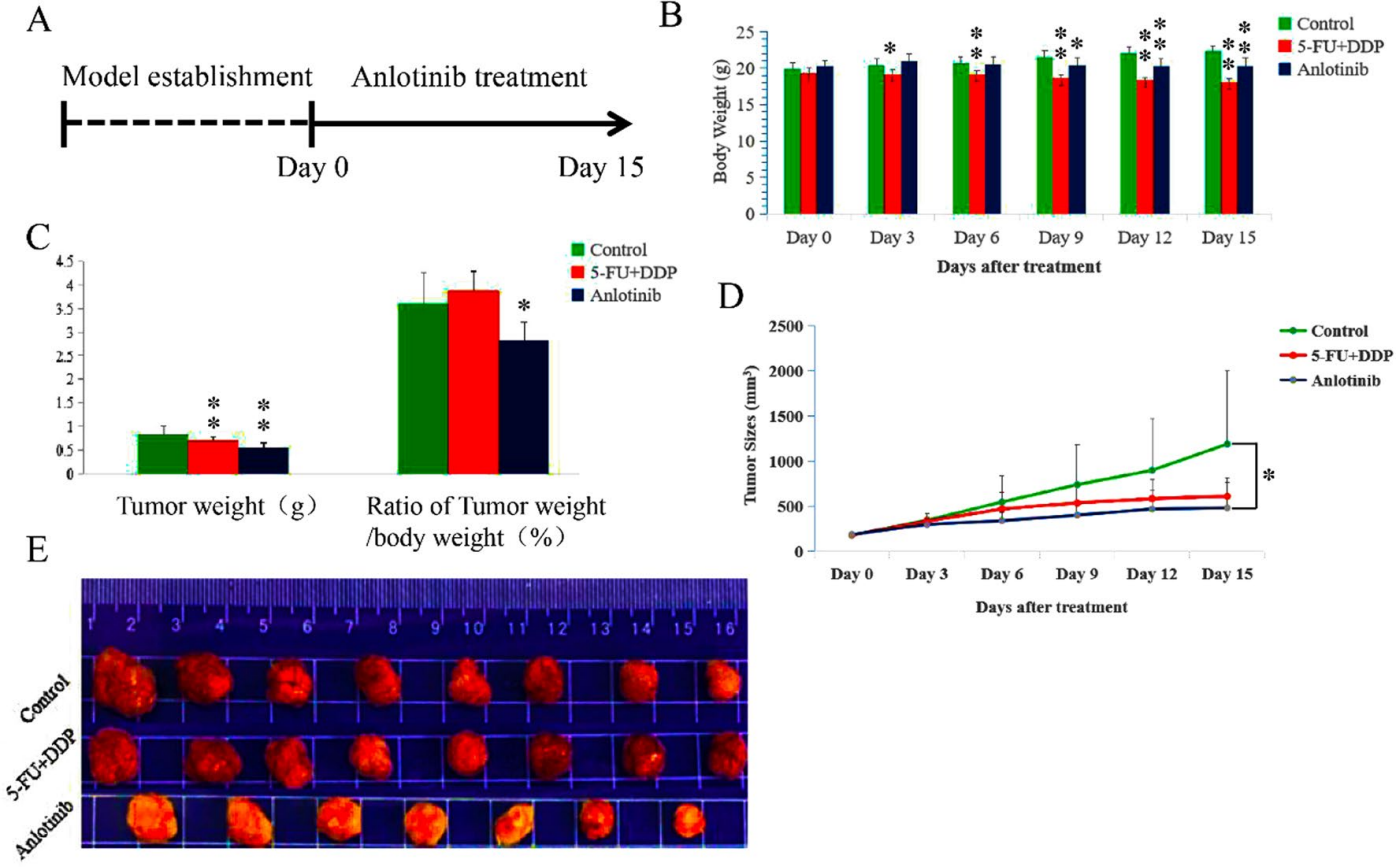
Anlotinib exhibits anticancer activity in TE-1 tumor cell xenograft mice.** A** Timeline of the experiment in vivo; **B** Anlotinib reduced mice body weight (for body weight, on day 9, *F* = 27.9, *P* < 0.05; on day 12 and 15, *F* = 52.6 and 70.6; *P* < 0.01;); **C** Anlotinib reduced tumor weight and ratio of tumor weight to body weight (%) (for tumor weight, *F* = 11.6, *P* < 0.01; for ratio of tumor weight to body weight, *F* = 8.5, *P* < 0.01); **D** Anlotinib reduced tumor size (on days 15; *F* = 4.1, *P* < 0.05); **E** Tumor tissues of different groups. Relative to the control, *P < 0.05, **P < 0.01; for the control and 5-FU + DDP groups, *n* = 8; for the anlotinib group, *n* = 7

To verify the antitumor activity in vivo, anlotinib was administered to TE- 1 tumor cell xenograft mice. The results indicated that anlotinib decreased tumor size (on day 15, F = 4.1, P < 0.05), tumor weight (on day 15, F = 11.6, P < 0.01), body weight (on day 9, F = 27.9, P < 0.05; on day 12 and 15, F = 52.6 and 70.6; P < 0.01), and the ratio of tumor weight to body weight (F = 8.5, P < 0.01) relative to the control; anlotinib slightly decreased the body weight and tumor size relative to 5-FU + DDP (Fig. 1A–E).

### Anlotinib decreases viability of TE-1 cells

**Fig. 2:**
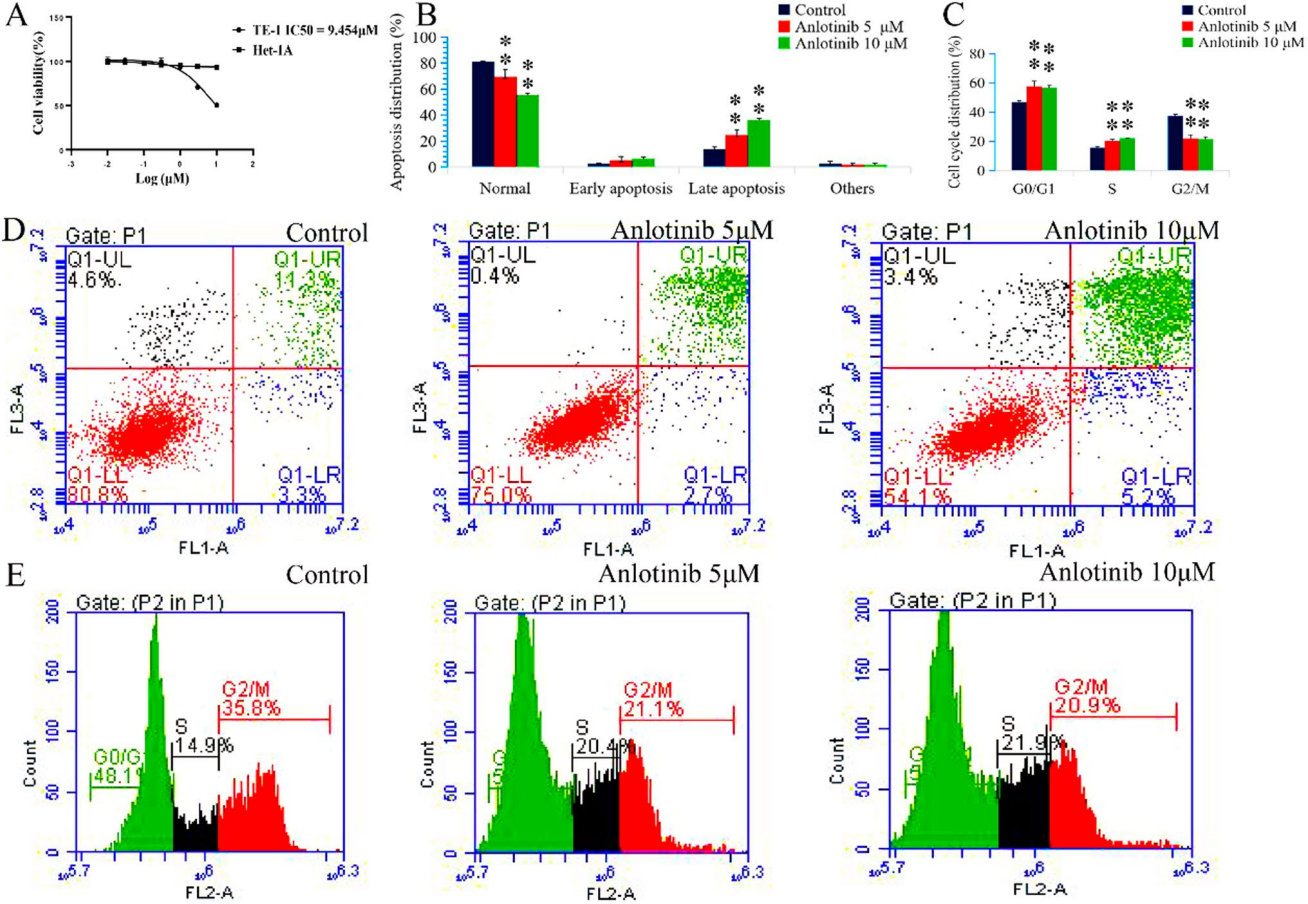
Anlotinib’s effects on cell viability, cell apoptosis, and cell cycle in TE-1 cells. **A** The growth curve of TE-1 and HET‑1A cells with anlotinib treatment; **B** and **D** The percentages of apoptotic TE-1 cells in late phase with anlotinib treatment (*F* = 42.5, *P* < 0.01); **C** and **E** The percentage of TE-1 cells in the G0/G1, S and G2/M phase with anlotinib treatment (*F* = 19.1, 37.8, 74.9; *P* < 0.01). Data are shown as the mean ± SD; relative to the control, ^**^P < 0.01, *n* = 3

After treatment with anlotinib (0.01, 0.03, 0.1, 0.3, 1, 3, and 10 μM), the TE- 1 cell growth curve decreased significantly with increasing dose, indicating that the cell viability declined in a dose-dependent manner. The 50% growth-inhibitory concentration (IC50) after anlotinib treatment was 9.454 μM for 24 h in TE- 1 cells (Fig. 2A). To determine whether anlotinib exerted inhibitory effects on normal cells, HET‑1A cells were also treated with anlotinib at the same concentrations with TE- 1 cells. The results demonstrated HET‑1A cells exhibited no significant differences after anlotinib treatment (Fig. 2A).

### Anlotinib induces apoptosis and cell cycle arrest in TE-1 cells

TE-1 cells were treated with anlotinib in doses of 5 or 10 μM, and a flow cytometry assay was used to measure the percentage of apoptotic cells. Relative to the control results, the percentages of late apoptotic cells were enhanced after anlotinib treatment (F = 42.5, P < 0.01) (Fig. 2B, D). We also studied cell cycle changes after anlotinib treatment. Interestingly, the results showed anlotinib arrested TE- 1 cells in G0/G1, S phase (F = 19.1, 37.8; P < 0.01) and prevented TE- 1 cells entering G2/M phase (F = 74.9, P < 0.01), (Fig. 2C, E).

### Anlotinib inhibits TE-1 cells migration and proliferation

**Fig. 3:**
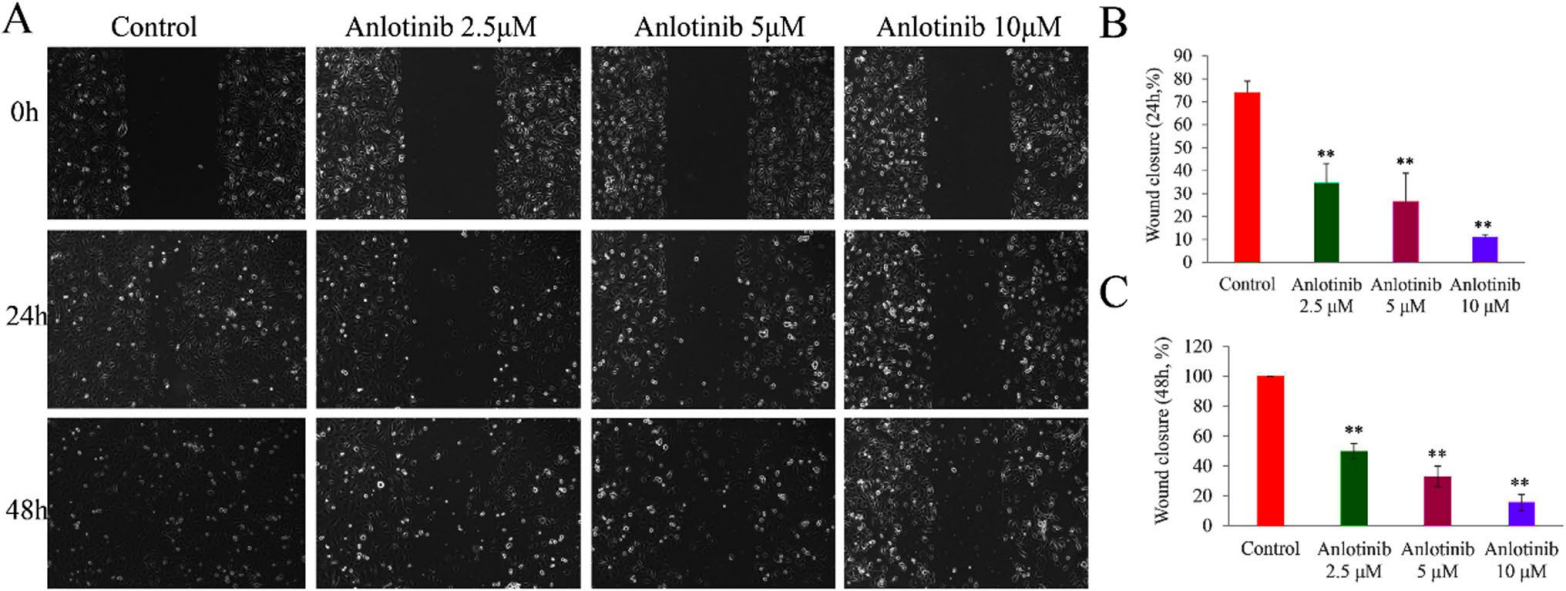
Anlotinib decreases wound closure of TE-1 cells in vitro. **A**–**C** Wound healing assays showed the migration of TE-1 cells treated with anlotinib (2.5, 5, 10 μM), and the percentage of wound closure was greatly inhibited after anlotinib treatment for 24, or 48 h, (*F* = 35.8, 156.7; *P* < 0.01). Data are shown as the mean ± SD; relative to the control, ^**^P < 0.01, *n* = 3

**Fig. 4:**
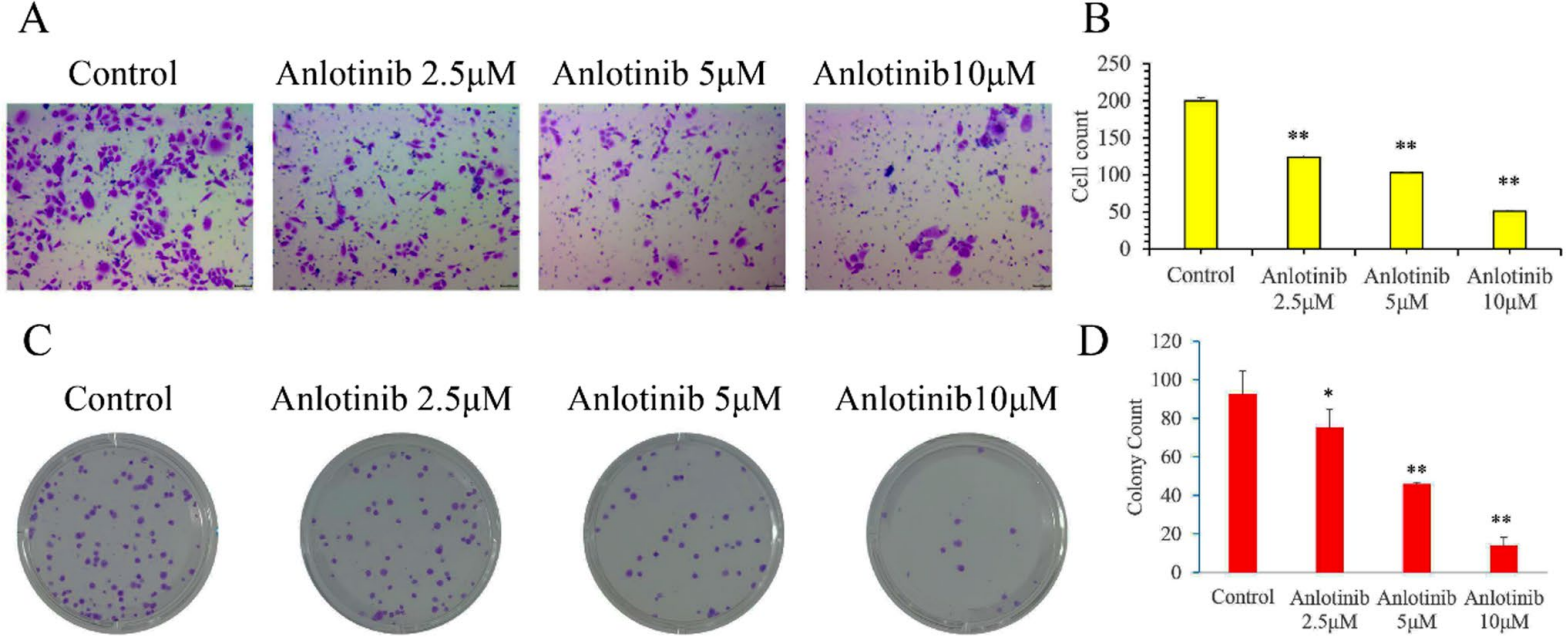
Anlotinib decreases migrated cell count and colony formation of TE-1 cells in vitro. **A**, **B** Transwell assays showed the migration of TE-1 cells treated with anlotinib (2.5, 5, 10 μM), and the extent of transwell migration was quantified by counting the stained cells, the migrated cell count was notably decreased after anlotinib treatment (*F* = 1316.3; *P* < 0.01). **C**, **D** Colony formation of TE-1 cells was also performed and it was decreased greatly by anlotinib in a dose of 2.5, 5 or 10 μM (*F* = 55.5, *P* < 0.01)

Subsequently, we studied anlotinib’effects on the migration of TE- 1 cells.Treatment with anlotinib (2.5, 5, or 10 μM) significantly decreased the percentage of wound closure in TE- 1 cells at 24 and 48h (F = 35.8, 156.7; P < 0.01) (Fig. 3A–C). Furthermore, TE-1 cells exhibited obvious lower migration through the membranes in the Transwell assays after anlotinib treatment (2.5, 5, or 10 μM), and the migrated cell counts were obviously decreased (F = 1316.3; P < 0.01) (Fig. 4A, B). Notably, colony formation of TE- 1 cells were also greatly inhibited after treatment of anlotinib (2.5, 5, or 10 μM), indicating that anlotinib inhibits TE- 1 cells proliferation (F = 55.5, P < 0.01) (Fig. 4C, D).

### Anlotinib affects the transcriptome in TE-1 cells

**Fig. 5:**
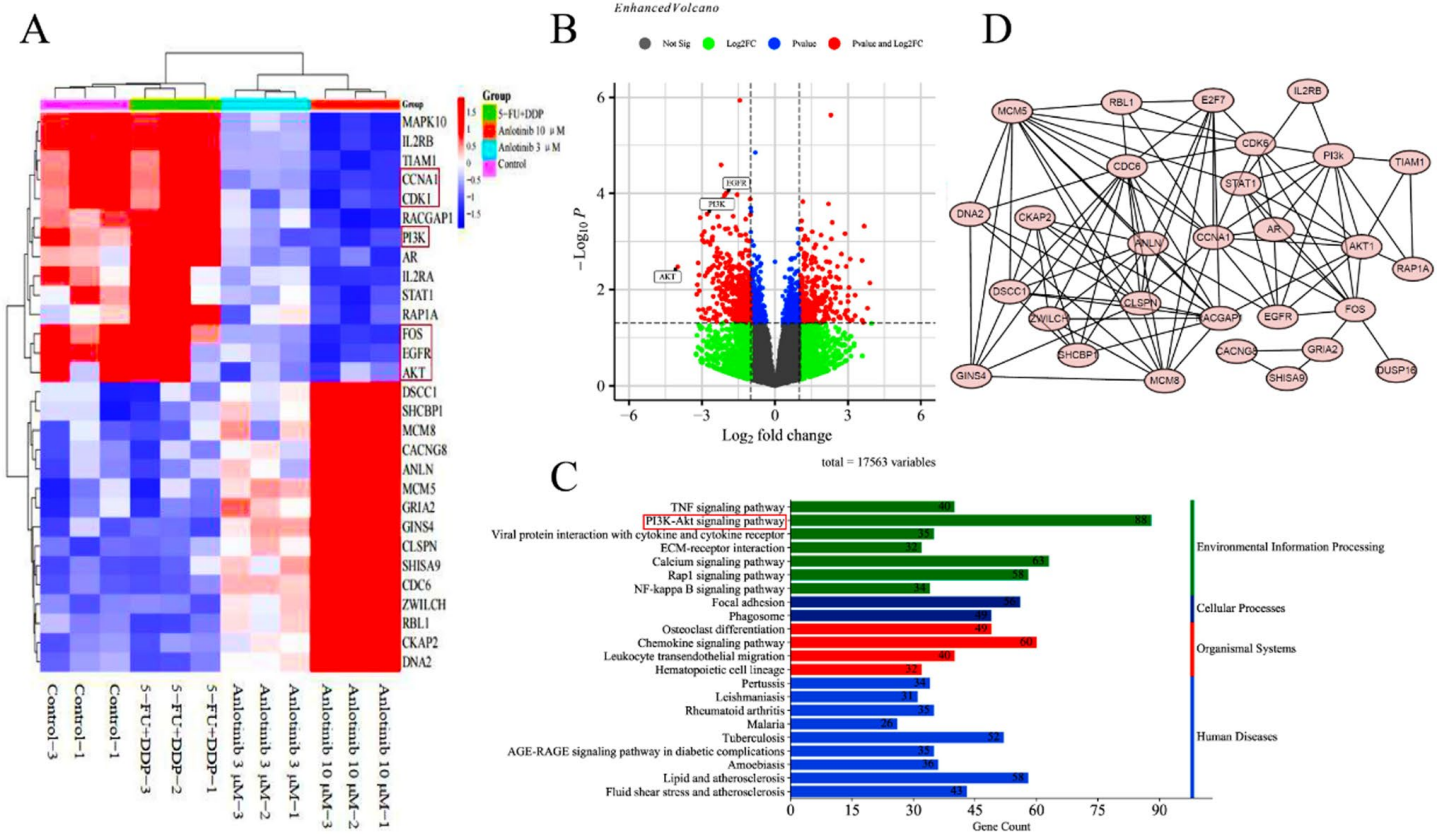
Bioinformatics analysis in transcripts in TE-1 cells with anlotinib treatment. **A** Heatmap showed the top 29 altered gene expressions in TE-1 cells after anlotinib treatment; **B** Enhanced Volcano exhibited altered gene expressions among 17,653 variables in TE-1 cells after anlotinib treatment based on adjusted *P* < 0.05 and fold-change = 2; **C** KEGG enrichment analysis uncovered PI3K/Akt signaling pathway was the most negatively enriched in TE-1 cells after anlotinib treatment. **D** PPI network showed Akt and CCNA1 were the two central genes in TE-1 cells affected by anlotinib treatment. Relative to control, *n* = 3

We used RNA-seq to analyze the effects of anlotinib treatment on transcriptional alteration in the TE- 1 cells. Heatmap exhibited the top 29 greatly altered gene expressions, the negative regulated gene expressions, such as PI3K, AKT, FOS, CCNA1 and CDK1 were involved in cell proliferation and cell cycle regulation (Fig. 5A). Enhanced volcano based on altered gene expressions revealed PI3K, AKT and EGFR were significantly down regulated among 17,653 variables (adjusted P < 0.05, fold-change = 2.0) (Fig. 5B); KEGG enrichment analysis uncovered PI3K-Akt signaling pathway was the most negatively enriched pathway, suggesting that anlotinib treatment suppresses the activity of the PI3K-Akt signaling pathway, which plays a crucial role in various aspects of cell proliferation, survival, and metabolism (Fig. 5C). PPI network was made using 30 functional related genes by Cytoscape software based on the top 200 extensively altered gene expressions matched with String database. The results demonstrated AKT and CCNA1 were the two central genes which interacted with other genes to regulate cell proliferation and cell cycle regulation (Fig. 5D).

### Anlotinib regulates cyclin A1 and cyclin B1/CDK1, Bax/Bcl-2 and PI3K/AKT signaling pathway in TE-1 cells

**Fig. 6:**
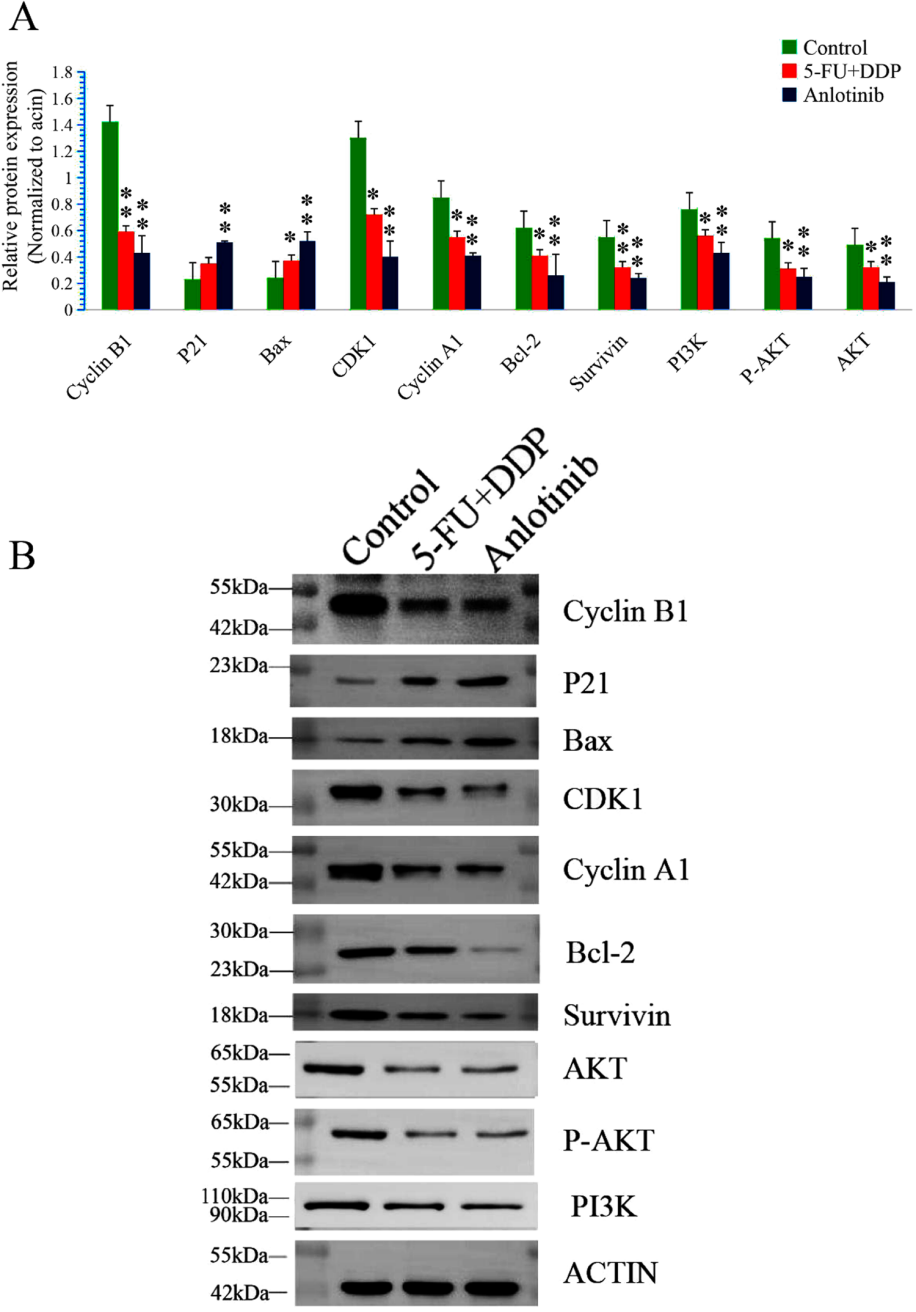
Anlotinib regulates cyclin A1 or cyclin B1/ CDK1, Bax/Bcl-2 and PI3K/Akt signaling pathways in TE-1 cells. Western blot analysis: **A** Anlotinib down regulated expressions of cyclin A1, cyclin B1, CDK1, Bcl-2, PI3K, Akt, p-Akt and survivin (*F* = 20.27, 24.11, 7.38, 9.03, 10.25, 9.62, 9.80 and 41.86, respectively, *P* < 0.01); up regulated expressions of P21, and Bax (*F* = 9.27 and 15.26 respectively, *P* < 0.01); **B** Images of western blotting. Data are shown as the mean ± SD; relative to the control, *P < 0.05, **P < 0.01; *n* = 3

To confirm results of the transcriptional alteration, western blotting was furthered performed to detect cell cycle related, apoptosis related and proliferation related proteins. After treatment with anlotinib, the expressions of cyclin A1, cyclin B1, CDK1, Bcl-2, PI3K, Akt, p-Akt and surviving was down regulated (F = 20.27, 24.11, 7.38, 9.03, 10.25, 9.62, 9.80 and 41.86, respectively, P < 0.01), while the expressions of P21 and Bax was up regulated (F = 9.27 and 15.26 respectively, P < 0.01) (Fig. 6A and B).

## Discussion

In China, approximately 240,000 new cases of EC are diagnosed each year, with EC ranking as the fourth leading cause of cancer-related mortality [13–15]. Many patients receive diagnoses at advanced stages, leading to 5-year survival rate of merely 5%. The discovery of new medicines for treating EC is an imperative need. Current studies on anlotinib have mostly focused on its anticancer activity against advanced non-small-cell lung cancer [7]. Jingzhen Shi combined anlotinib with chemoradiotherapy to treat EC with good achievements [16]. These results have inspired us to investigate the underlying mechanisms behind anlotinib's effects on EC.

In our study, anlotinib demonstrated excellent antineoplastic activity against EC in vivo, with less body weight loss in comparison to the 5-FU + DDP treatment. This effect was corroborated by the outcomes observed in the TE-1 cell line, where anlotinib exhibited an IC50 value of 9.454 μM. Furthermore, anlotinib induced TE- 1 cell apoptosis and notably arrested the cell cycle in the S phase, thereby inhibiting migration and proliferation in a dose-dependent manner. These findings indicate that Anlotinib has potential therapeutic capabilities against esophageal cancer, exhibiting significant anti-tumor effects both in vivo and in vitro. Furthermore, Anlotinib effectively inhibits the migration and proliferation of esophageal cancer cells by inducing apoptosis and arresting the cell cycle, providing important evidence for its further research and clinical application as an anti-cancer treatment.

The PI3K/Akt signaling pathway is a crucial cellular signaling cascade involved in regulating various biological processes such as cell survival, proliferation, differentiation, and metabolism [17, 18]. Its name derives from two key proteins involved in the pathway: phosphoinositide 3-kinase (PI3K) and protein kinase B (Akt), also known as protein kinase B activated kinase (PKB). PI3K is an enzyme that catalyzes the conversion of phosphatidylinositol 4,5-bisphosphate (PIP2) to phosphatidylinositol 3,4,5-trisphosphate (PIP3) on the cell membrane [19, 20]. Upon activation, PIP3 can bind and activate Akt, thereby initiating downstream signaling cascades. Akt, a critical regulatory protein in the PI3K/Akt pathway, promotes cell survival and proliferation while inhibiting apoptosis. Akt exerts its effects by phosphorylating various cellular factors, cell cycle proteins, and transcription factors, thereby modulating cellular physiology. Aberrant activation of the PI3K/Akt signaling pathway is closely associated with the onset and progression of various diseases, including cancer [21, 22]. PI3K/Akt signaling pathway plays a crucial role in the proliferation, survival, and invasion of tumor cells [23, 24] including TE- 1 cells [25–27]. The activation of PI3K promotes Akt to be phosphorylated intop-Akt, p-Akt acts on its downstream proteins to enhance TE-1 cell survival, proliferation and invasion. The PI3K/Akt signaling pathway can also participate in cell survival and inflammatory responses by activating NF-κB (nuclear factor-kappa B). NF-κB is a transcription factor that regulates the expression of various genes, including those associated with cell survival, proliferation, and immune responses. The excessive activation of the PI3K/Akt signaling pathway is closely associated with the occurrence and development of various diseases, including cancer, diabetes, and neurological disorders. Therefore, this signaling pathway has become a crucial target for drug development, and drugs targeting its abnormal activation are being investigated for the treatment of diseases such as cancer. Our findings indicated that anlotinib treatment notably downregulated PI3K, Akt, and p-Akt, aligning with the results of other studies [9, 10].

Survivin plays a significant role in regulating cell division, apoptosis (programmed cell death), and cell survival. It is a member of the inhibitor of apoptosis (IAP) family of proteins, which are characterized by their ability to inhibit apoptosis and promote cell survival [28, 29]. Survivin is overexpressed in various types of human tumor cells, including lung cancer, breast cancers, EC, as well as TE-1 cells, as indicated by our research. The suppression of survivin promotes tumor cells apoptosis and enhances radiosensitivity of esophageal cancer cells [30–32]. Besides, Bax serves as an apoptosis promoter, while Bcl-2, an important homolog of Bax, plays the inverse effects of Bax [33, 34]. Our results demonstrated that anlotinib treatment significantly promoted TE- 1 cells apoptosis through suppression of survivin, Bcl-2 and enhancement of Bax expressions. By understanding how Anlotinib regulates key genes such as survivin, Bcl-2, and Bax to promote apoptosis in TE-1 cells, we can gain deeper insights into its mechanism of action in anticancer processes, which can guide further clinical research and the development of more effective treatment strategies.

Cyclin A1 is the product of CCNA1 gene expression. Except Akt as one of the two central proteins affectd by anlotinib treatment, Cyclin A1 was the other central protein which interacting with other proteins to regulate cell cycle. Cyclin A1 which working together with cyclinB1 to promote S to G2/M phase transition. The combination of cyclin A1 or cyclinB1 to CDK1 triggers cell cycle into mitosis [35–37]. P21 is currently recognized as a potent universal CDK inhibitor which forms complexes with CDKs and cyclins to arrest cell cycle [38, 39]. Anlotinib negatively regulated cyclin A1, cyclin B1 and CDK1 expressions while positively up regulated P21 expression in TE- 1 cells. These observations elucidate the mechanism underlying its effects on cell cycle arrest.

This article has some shortcomings. Firstly, although the study elucidated the negative regulation of the PI3K/Akt signaling pathway by Anlotinib, the underlying mechanisms of this regulation have not been fully explored. Further mechanistic studies are needed to analyze the molecular pathways involved and confirm the observed effects. Secondly, TE-1 cells may not fully represent the heterogeneity of esophageal cancer. Including other esophageal cancer cell lines or samples originating from patients could provide a more comprehensive understanding of the effects of Anlotinib in different molecular subtypes of the disease.

## Conclusions

Anlotinib can induce apoptosis and cell cycle arrest, inhibit migration and proliferation of TE- 1 cells by negatively regulating PI3K/Akt signaling pathway, and consequently regulating expressions of apoptosis- related and cell-cycle-related proteins. The detailed underlying mechanism may be further elucidated in future research.

## Data Availability

The datasets generated during and/or analyzed during the current study are available from the corresponding author upon reasonable request.
